# Optimization of hypovascular liver lesion detectability in dual-energy CT using deep learning image reconstruction: a phantom study for potential iodine dose reduction

**DOI:** 10.1186/s41747-026-00759-2

**Published:** 2026-07-01

**Authors:** Marianna Gulizia, Clarisse Dromain, Laura Haefliger, Hussayn Chettab, Christine Chevallier, Anaïs Viry

**Affiliations:** 1 Department of Radiology and Interventional Radiology, Lausanne University Hospital and University of Lausanne, Lausanne, Switzerland; 2 Institute of Radiation Physics, Lausanne University Hospital and University of Lausanne, Lausanne, Switzerland

**Keywords:** Contrast media, Deep learning, Liver neoplasms, Phantoms (imaging), Tomography (x-ray computed)

## Abstract

**Objective:**

To determine the optimal low-keV level using deep learning image reconstruction (DLIR) that maximizes lesion detectability, and to assess the potential for iodinated contrast media (ICM) reduction based on detectability improvements across varying patient body habitus.

**Materials and methods:**

An abdominal phantom was scanned using a standard thoraco-abdomino-pelvic dual-energy computed tomography (DECT) protocol during the portal venous phase, with three rings inserted simulating different body habitus. Virtual monoenergetic images (VMI) were reconstructed from 40 to 70 keV in 10 keV increments using adaptive statistical iterative reconstruction-V (ASIR-V) 50% and high-strength DLIR (DLIR-H). Contrast enhancement was quantified, spatial resolution was evaluated with the task-based transfer function, and noise characteristics were analyzed using the noise power spectrum. Low-contrast lesion detectability (5–10 mm) was assessed using an anthropomorphic model observer.

**Results:**

Compared to ASIR-V, DLIR-H provided equivalent contrast, reduced image noise, and improved spatial resolution. All lesion sizes with DLIR-H were technically detectable under all conditions. The reconstruction at 40 keV demonstrated the highest detectability of hypovascular lesions under all conditions. However, a decrease in detectability was observed in the large phantom relative to the small and medium phantoms, resulting in a reduced theoretical potential for iodine dose reduction. The theoretical potential for iodine dose reduction using 40 keV with DLIR-H is at least 31.3% based on the phantom-based model.

**Conclusion:**

Under phantom conditions, 40 keV with DLIR-H shows superior detectability of hypovascular lesions under all conditions, suggesting the theoretical possibility of reducing iodine load by up to 31.3%, based on modeled detectability performance.

**Relevance statement:**

Based on a phantom-derived model, the combination of 40-keV VMI reconstruction with DLIR-H suggests the potential for more than 30% ICM reduction in oncologic body CT, a finding that warrants confirmation in clinical studies.

**Key Points:**

- Based on a phantom-derived model 40 keV VMI with DLIR-H achieved the highest detectability of hypovascular liver lesions.
- This approach enabled a 31.3% ICM volume reduction.
- Larger body habitus limits ICM volume reduction optimization margins.

**Graphical Abstract:**

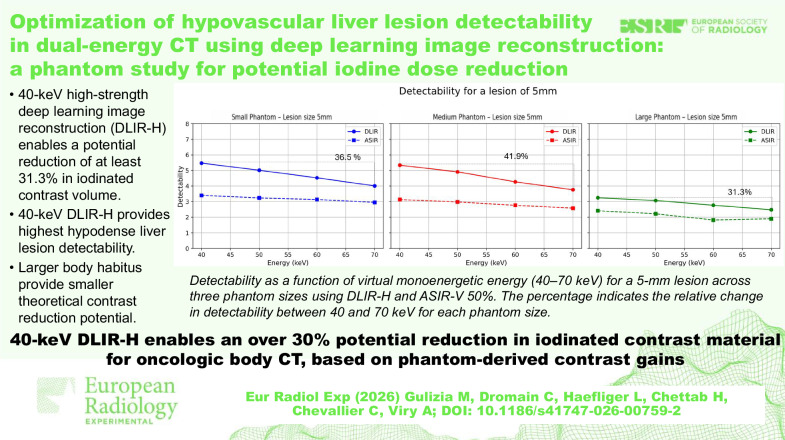

## Background

Medical imaging plays a pivotal role in oncology, not only in the initial diagnosis but also in the longitudinal monitoring of treatment and follow-up [1, 2]. Among imaging modalities, contrast-enhanced thoraco-abdomino-pelvic CT is widely used for oncologic imaging due to its rapid acquisition time, widespread availability, and comprehensive whole-body coverage [3, 4]. Ensuring high diagnostic quality and lesion detectability is critical, particularly when evaluating subtle findings such as small liver metastases, which can significantly impact therapeutic decisions.

Recent advances in imaging technology, particularly dual-energy CT (DECT), have significantly improved contrast resolution through low-keV virtual monoenergetic image (VMI) reconstructions, thereby enhancing lesion conspicuity and detectability [5, 6]. However, lower keV levels are inherently associated with increased image noise, which may offset these benefits and compromise diagnostic accuracy. To overcome this limitation, deep learning image reconstruction (DLIR) has emerged as a promising solution providing image noise reduction [7, 8]. In fact, as reported by Greffier et al, high-strength DLIR (DLIR-H) improved noise texture by 10% compared to the adaptive statistical iterative reconstruction-V at 50% strength (ASIR-V) [9]. Additionally, the combined application of low-keV reconstructions and DLIR has demonstrated an increase of 60 ± 4% (mean ± standard deviation) in detectability for low-contrast liver lesions [9].

This improved contrast at low-keV settings also importantly opens the door to reducing the amount of iodinated contrast media (ICM) required. Iodine volume reduction can enhance patient safety by lowering the risk of contrast-related complications, including contrast-induced nephropathy in patients with pre-existing renal impairment. It also helps reduce healthcare costs and minimizes environmental impact by decreasing the release of ICM into aquatic ecosystems.

However, any reduction in contrast media must be carefully balanced against the need to maintain adequate image quality for diagnostic accuracy. Preserving the detectability of low-contrast lesions, such as hypovascular liver lesions, is critical. Several clinical studies conducted on patients have empirically explored this potential by reducing the volume of ICM by 44% to 50% [10, 11]. However, no prior studies have assessed the potential ICM reduction across different body habitus.

The aim of this study is to determine the optimal low-keV setting for the detection of hypovascular liver lesions when combined with DLIR. In parallel, we aim to evaluate the feasibility of reducing the amount of ICM required for oncologic thoraco-abdominopelvic DECT examinations, while preserving the detectability of hypovascular liver lesions across a range of simulated patient body sizes. This dual approach seeks to inform personalized contrast dosing strategies that consider both image enhancement and patient-specific anatomical variability.

## Methods

The study was approved by the local ethics committee (CER-VD n°2025-00567). In this experimental phantom study, an anthropomorphic abdominal phantom was designed based on real patient imaging data, ensuring clinically relevant anatomical and contrast conditions.

### Retrospective analysis of real-world oncologic DECT examinations

To replicate clinically relevant conditions, we retrospectively reviewed 90 thoraco-abdomino-pelvic DECT examinations performed in the portal venous phase for oncologic follow-up at our institution, all of which included confirmed liver metastases.

All DECT examinations were performed using a 256-detector row scanner (Revolution CT, General Electric Healthcare). DECT was conducted with Gemstone Spectral Imaging, using ultra-fast kV switching between 80 kVp and 140 kVp. The detailed scanning parameters are listed in Table 1.

**Table 1:** DECT parameters

Parameters	DECT
Tube voltage (kVp)	80–140
Pitch	0.992
Collimation (mm)	80 × 0.625
SFOV (mm)	500
Matrix size (pixels)	512 × 512
Gantry rotation time (s/rotation)	0.6
Slice thickness (mm)	2.5
Slice increment (mm)	2
Kernel	Standard
Reconstruction	ASIR-V 50%, DLIR-H
DECT reconstruction (KeV)	40, 50, 60, 70

*ASIR-V* Adaptive statistical iterative reconstruction-V, *DLIR-H* High-strength deep learning image reconstruction, *DECT* Dual-energy computed tomography, *SFOV* Scan field of view

At our institution, the standard thoraco-abdomino-pelvic DECT protocol for oncologic imaging consists of a single acquisition performed during the portal venous phase, 75 s after intravenous contrast administration, without the use of bolus tracking. Contrast injection is administered *via* an automatic power injector (CT Exprès® 3D Contrast Media Delivery System, Bracco), using iohexol at a concentration of 300 mg iodine/mL (Accupaque 300, GE Healthcare, Nycomed). The ICM is injected into an antecubital vein at a rate of 2–4 mL/s, depending on the total volume, which is calculated based on patient weight using the following protocol: 1 mL/kg (equivalent to 0.3 g I/kg of body weight). The performance of DLIR combined with low-keV reconstruction was assessed across three simulated body habitus categories, defined according to the World Health Organization body mass index (BMI) classification: underweight (BMI < 18.5 kg/m²), normal weight (BMI 18.5–24.9 kg/m²), and overweight (BMI 25–30 kg/m²). To replicate realistic anatomical conditions, morphometric parameters from 30 oncologic patients in each BMI category, including height, weight, and abdominal anterior-posterior and transverse diameters measured at the level of the largest cross-sectional area, were retrospectively extracted from clinical data and used to determine the appropriate extension ring configuration for the anthropomorphic phantom (Table 1).

To ensure clinically relevant dose conditions for phantom acquisitions, median computed tomography dose index values were calculated for each BMI group: 6.2 ± 0.6 mGy for underweight, 8.9 ± 3.5 mGy for normal weight, and 12.3 ± 2.5 mGy for overweight patients, corresponding to tube currents of 200, 300, and 405 mA, respectively.

### Anthropomorphic abdominal phantom design

**Fig. 1:**
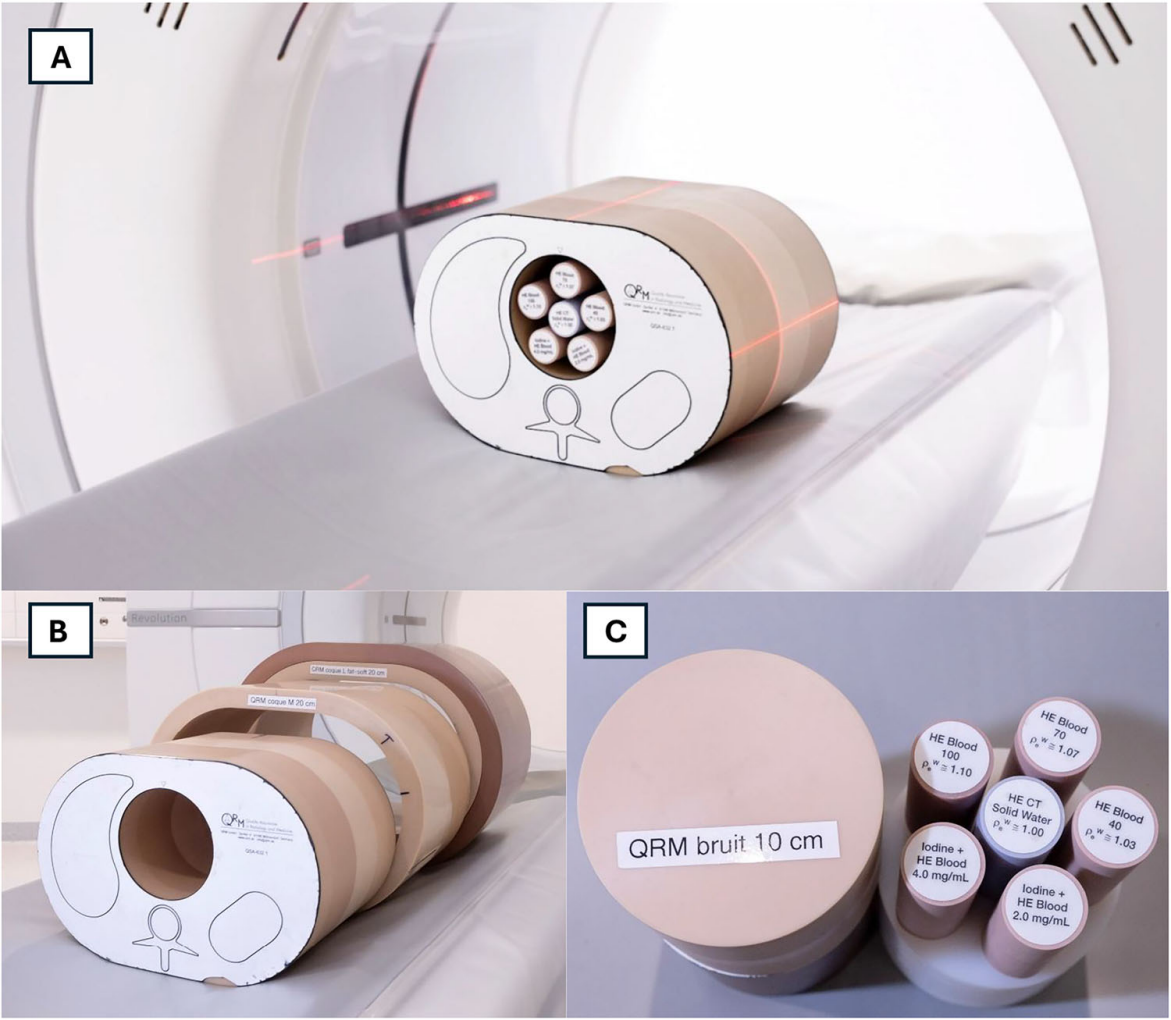
Experimental phantom setup for contrast detectability assessment. **A** Overview of the QRM anthropomorphic body phantom positioned on the computed tomography table. **B** Two extension rings used for the configuration of the medium and large-sized body phantom: a 2.5-cm-thick ring simulating soft tissue, and a 5-cm-thick ring representing a combination of soft tissue and adipose tissue attenuation. **C** Two dedicated modules were used: one module designed for task-based transfer function measurements, and a second module containing cylindrical inserts filled with a blood-equivalent solution supplemented with iodine at different concentrations, surrounding a central solid-water reference insert, positioned using a polyethylene holder for contrast and attenuation assessment

An anthropomorphic abdominal phantom (QRM, a PTW company), measuring 30 × 20 cm, was used to evaluate the detectability of hypovascular liver lesions across various VMI reconstructions and reconstruction algorithms (Fig. 1). This base phantom replicates the x-ray attenuation properties of an underweight patient. To simulate additional body habitus categories, two extension rings were added: a 2.5-cm-thick ring simulating soft tissue, and a 5-cm-thick ring representing a combination of soft tissue and adipose tissue attenuation.

Dedicated image quality modules were inserted into the phantom to assess contrast, noise, and spatial resolution. These modules were specifically designed to replicate the attenuation characteristics of hypovascular liver lesions during the portal venous phase.

Contrast was evaluated using cylindrical inserts filled with a blood-equivalent solution supplemented with iodine at concentrations of 0, 2, and 4 mg I/mL (Sun Nuclear). The inserts were placed within the phantom using a polyethylene holder. Linearity between CT attenuation and iodine concentration across different keV levels was assessed using linear regression analysis. To ensure clinical relevance, attenuation values of the liver parenchyma and liver metastases were measured in images from patients within the BMI 18.5–24.9 range to determine appropriate insert concentrations for the phantom study. Root mean square errors were subsequently computed to determine the iodine concentrations that best mimic the parenchymal and metastatic attenuation profiles.

Spatial resolution was assessed using a dedicated module developed by QRM (QRM, a PTW company), consisting of a central rod with a diameter of 4 cm, embedded in a homogeneous background. Both the rod and the background were filled with iodine solutions, differing by 1.5 mg I/mL in concentration. Image noise was computed using a homogeneous insert.

Each of the three phantom configurations, simulating underweight, normal weight, and overweight body habitus, was scanned with the same clinical DECT protocol using the Revolution Apex scanner (GE Healthcare). To ensure comparability with clinical conditions, the noise index was individually adjusted for each phantom size to correspond to the median computed tomography dose index values obtained in the retrospective analysis of real-world oncologic DECT examinations for each BMI group. The display field of view was set at 320 mm, 370 mm, and 420 mm for the small, medium, and large phantoms, respectively, to simulate patient-specific field-of-view settings.

### Phantom image analysis

To assess the detectability of simulated hypovascular hepatic lesions in the context of oncologic CT imaging, a non-prewhitening model observer with an eye filter was employed. This mathematical observer model incorporates a human visual response function and evaluates lesion detectability based on key image quality metrics such as contrast, noise, and spatial resolution.

#### Contrast

The contrast of simulated hypovascular lesions was calculated as the difference between the attenuation values of the two iodine concentrations that best mimic the parenchymal and metastatic attenuation profiles.

#### Spatial resolution

To evaluate spatial resolution, the transition between the iodine rod and the surrounding soft tissues was used to measure the task-based transfer function (TTF). The TTF metric is particularly suited to assess contrast-dependent spatial resolution in non-linear and non-stationary reconstruction methods, such as ASIR-V and DLIR [12]. The circular edge between the iodine rod and soft tissues was used to calculate edge spread functions. The TTFs were then calculated using the Fourier transform of the edge spread functions, and the radial mean TTF was normalized to 1.0 at zero spatial frequency. Spatial resolution was quantitatively compared across VMI energy levels, phantom sizes, and reconstruction algorithms using the spatial frequency at 50% as a reference index.

#### Noise

Noise was assessed using the noise power spectrum in accordance with the methodology previously described in Report 87 of the International Commission on Radiation Units and Measurements [13]. The noise power spectrum was calculated by placing 500 regions of interest, each measuring 60 × 60 mm², within the homogeneous module of the phantom. This approach allowed for a robust characterization of noise magnitude and frequency distribution, which are critical parameters for evaluating image quality, particularly in the context of advanced reconstruction techniques. The noise amplitude was calculated using the area under the noise power spectrum curve. Although the analysis was performed on a phantom, it provides valuable insight into the behavior of image noise under controlled conditions. These findings can be extrapolated to clinical imaging, as similar noise characteristics influence image quality and signal-to-noise performance in patients, especially across different body sizes.

#### Detectability of hypovascular lesion

The detectability of simulated hepatic lesions was assessed using a non-prewhitening model observer with eye filter (Eq. 1). This model is widely used to evaluate low-contrast detectability in CT imaging studies and in clinical routine [7, 9, 14–16]. It was previously validated against human observer performance in a preliminary step involving two human observers and demonstrated a very good correlation.

1 $${d^{\prime} }_{{NPWE}}=\sqrt{2\pi }\left\lfloor \Delta {HU}\right\rfloor \frac{{\int }_{0}^{{f}_{{ny}}}{S}^{2}\left(f\right){{TTF}}^{2}\left(f\right){{VTF}}^{2}\left(f\right){fdf}}{\sqrt{{\int }_{0}^{{f}_{{ny}}}{S}^{2}\left(f\right){{TTF}}^{2}\left(f\right){NPS}\left(f\right){{VTF}}^{4}\left(f\right){fdf}}}$$

where |ΔHU| is the absolute value of the contrast, f the radial spatial frequency, f_ny_ the Nyquist frequency and TTF and noise power spectrum were previously described. S represents the circular section of a lesion [17]. The lesion size ranged between 5 and 10 mm, as observed in the retrospective analysis of oncologic DECT examinations. VTF is the visual transfer function of the human eye described by Gang et al [16]. Detectability values typically ranged from 0 to infinity. A monotonic function can be used to link the detectability with the area under the receiver operating characteristic curve [18]. A detectability index (d’) between 1.2 and 1.9 corresponds to an area under the receiver operating characteristic curve between 0.8 and 0.9, which is generally considered good. A detectability index above 1.9 typically reflects excellent lesion conspicuity.

### Potential iodinated contrast media reduction

Studies employing a circulation phantom model have demonstrated a proportional relationship between the contrast-to-noise ratio and the iodine delivery rate [19–21], in order to evaluate the potential for reducing iodine load. In the present study, the contrast-to-noise ratio was substituted with detectability, a more comprehensive performance metric that incorporates both the size of the target object and the spatial resolution of the imaging system. To assess the potential for ICM reduction, the percentage difference in lesion detectability between the optimal keV level (as determined by quantitative analysis) and the clinical reference standard of 70 keV was calculated. This variation in detectability was used to estimate the gain in tissue iodine concentration with low-keV VMI while maintaining the same detectability in standard conditions (70 keV). This is illustrated by the following Eq. 2:

2 $${{{\rm{Potential}}}}\; {{{\rm{of}}}}\; {{{\rm{iodine}}}}\; {{{\rm{reduction}}}}( \% ):\frac{{d^{\prime} }_{{optimalkeV}}-\,{d^{\prime} }_{{ref}70{keV}}}{{d^{\prime} }_{{ref}70{keV}}}$$

### Statistical analysis

All analyses were performed using the Scipy (v1.9.0) package for Python (v3.8). Continuous data were presented as mean ± standard deviation. Linear regression was performed to confirm the relationship between the iodine concentrations of the inserts and the measured attenuation at different keV levels. The goodness of fit was evaluated using the correlation coefficient (r), with r ≥ 0.9 considered indicative of a strong linear relationship. As TTF50% is computed from the task-based transfer function using a high number of acquisitions per condition, its estimate does not incorporate measurement repeatability. Consequently, no standard deviations or statistical hypothesis testing could be performed, and results are reported descriptively. Instead, comparative analyses were conducted by evaluating trends across phantom sizes (small, medium, large) and reconstruction methods (ASIR-V and DLIR-H). For each phantom and reconstruction, TTF50% values were plotted as a function of energy (40–70 keV) to evaluate the slope of the TTF50%–keV relationship, providing a qualitative measure of the rate of improvement in spatial resolution with increasing energy.

The differences in detectability were expressed as percentage gain to facilitate comparison across phantom sizes and conditions. Due to the high number of acquisitions for each condition necessary to precisely compute the various metrics, no standard deviation can be calculated for the mathematical model observer.

## Results

### Retrospective analysis of real-world oncologic DECT examinations

The anterior-posterior and transverse diameter and mean BMI observed in the three patient groups are presented in Table 2.

**Table 2:** Patient characteristics

Patient group	Weight (kg)	Height (cm)	BMI (kg/m^2^)	Anterior-posterior diameter (cm)	Transverse diameter (cm)
BMI < 18 (small)	49.6 ± 6.6	168.4 ± 10.1	17.4 ± 0.4	29.3 ± 2.6	18.78 ± 2.1
BMI 18.5–25 (medium)	66.6 ± 12.6	172 ± 9.9	22.2 ± 2.5	34.4 ± 3.8	22.7 ± 3.6
BMI 25–30 (large)	85.0 ± 7.8	175.4 ± 7.5	27.6 ± 2.5	37.8 ± 2.4	26.4 ± 2.5

Data are presented as mean ± standard deviation

*BMI* Body mass index

The diameters observed in the patient were similar to those of the three phantom sizes (30 × 20 cm, 35 × 25 cm, 40 × 30 cm). A strong linear correlation (R > 0.9) was observed between CT attenuation and the iodine concentrations of the three blood-mimicking inserts across all VMI energy levels. The iodine concentrations that best approximated the mean attenuation values of liver metastases and liver parenchyma were determined to be 0.45 mg/mL and 2.0 mg/mL, respectively. A summary of mean attenuation values for each VMI level is provided in Table 3.

**Table 3:** Mean HU attenuation values for metastases and liver parenchyma in patients at varying keV settings with deep learning image reconstruction

keV	Metastases	Insert blood-iodine 0.45 mg/mL	Liver parenchyma	Insert blood-iodine 2 mg/mL
40	90.6 ± 21.3	107.72 ± 17.2	232.3 ± 20.2	243.20 ± 13.4
50	66.9 ± 16.0	80.69 ± 12.4	169.6 ± 15.0	169.20 ± 10.9
60	52.3 ± 12.2	65.07 ± 9.1	129.4 ± 11.4	123.90 ± 7.1
70	43.2 ± 10.0	53.97 ± 7.8	105.0 ± 9.1	95.80 ± 6.4
80	37.8 ± 8.5	47.47 ± 5.5	90.1 ± 7.7	78.10 ± 4.4
120	28.2 ± 6.3	39.83 ± 3.8	64.2 ± 5.5	57.20 ± 3.1

Data are presented as mean ± standard deviation

### Phantom study

#### Contrast lesion

**Fig. 2:**
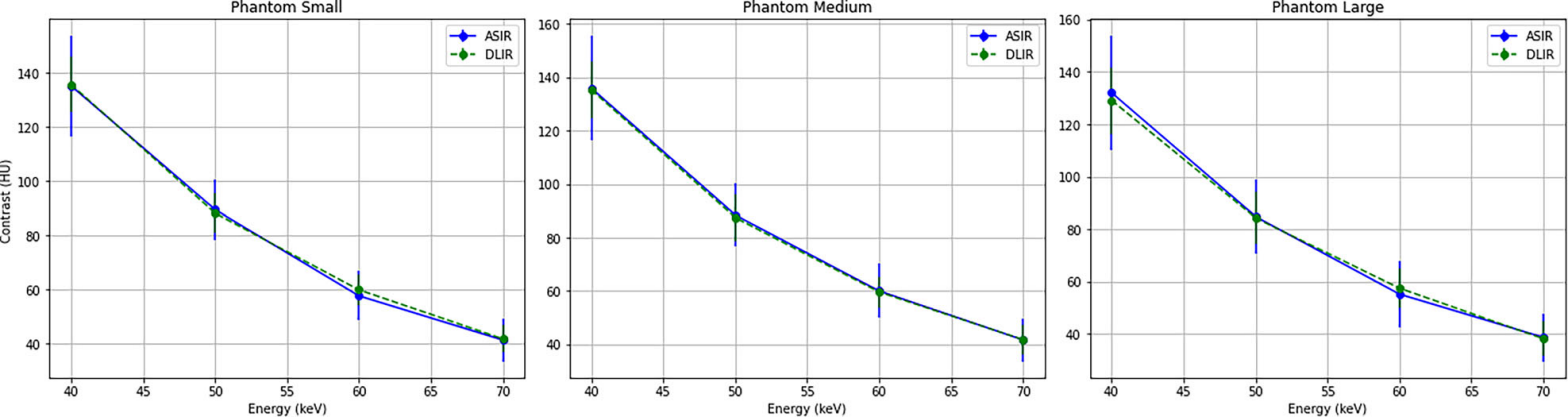
Comparison of contrast values between ASIR-V and DLIR across three phantom sizes (small, medium, large) at different energy levels (40–70 keV). ASIR, Adaptive statistical iterative reconstruction; DLIR, Deep learning image reconstruction

**Fig. 3:**
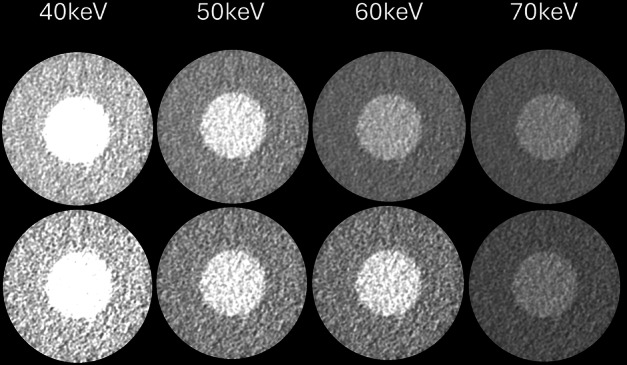
Qualitative comparison of image contrast and noise texture of QRM module across virtual monoenergetic image reconstructions (40, 50, 60, and 70 keV) and reconstruction algorithms (ASIR-V and DLIR-H). Top row: DLIR-H reconstructions. Bottom row: ASIR-V 50% reconstructions. ASIR, Adaptive statistical iterative reconstruction; DLIR-H, High-strength deep learning image reconstruction

Contrast values across energy levels for the three phantom sizes are presented in Fig. 2. As expected, contrast decreased progressively with increasing VMI energy (Fig. 3). The highest contrast was observed at 40 keV, showing a fourfold increase compared to 70 keV images. Similar contrast was noted between the two reconstruction algorithms, ASIR-V 50% *versus* DLIR-H. Additionally, contrast measurements were consistent across the different phantom sizes, indicating that body habitus had minimal impact on contrast enhancement.

#### Spatial resolution

**Fig. 4:**
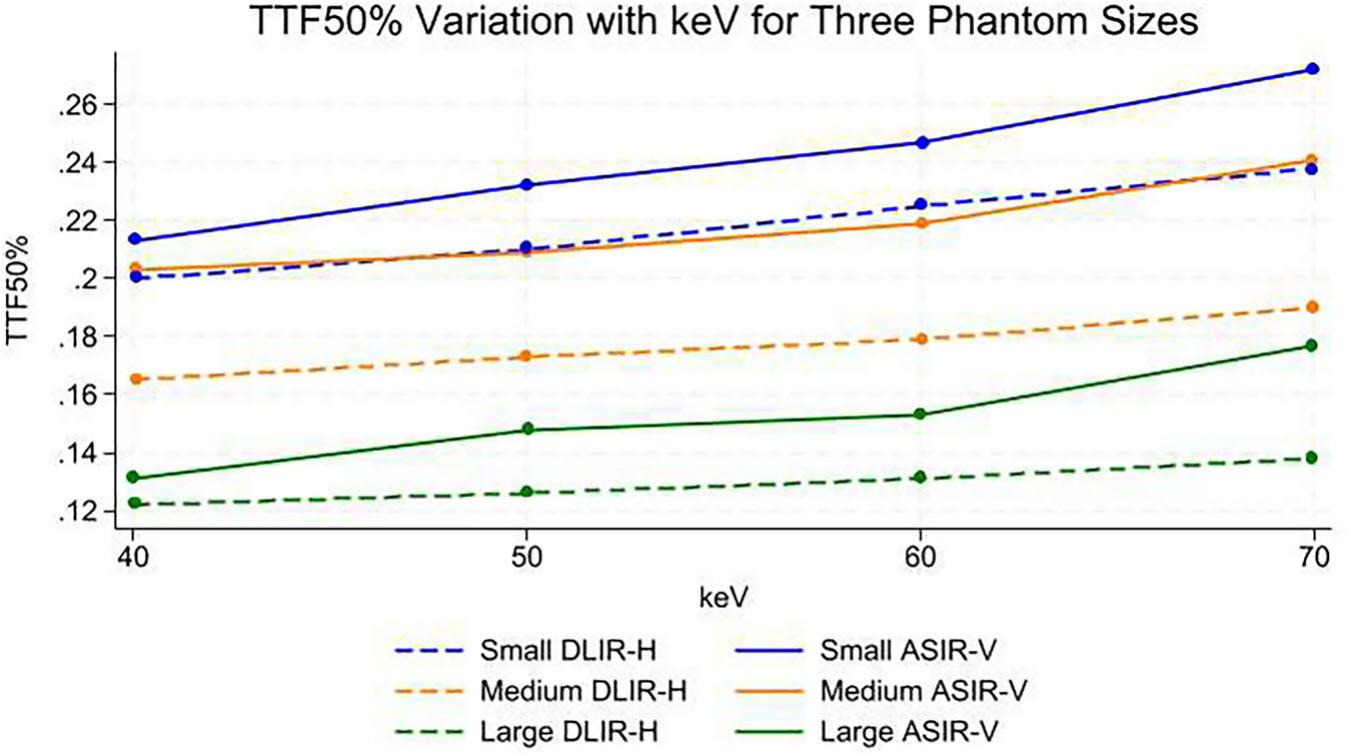
TTF50% variation with keV for three phantom sizes reconstructed using ASIR-V and DLIR-H. ASIR, Adaptive statistical iterative reconstruction; DLIR-H, High-strength deep learning image reconstruction; TTF, Task-based transfer function

TTF values at 50% are summarized in Supplement S1 (online) and Fig. 4 for the different VMI energy levels, reconstruction algorithms, and phantom sizes. For all phantom sizes, both reconstruction techniques showed a progressive improvement in TTF50% with increasing keV. ASIR-V 50% provided slightly higher spatial resolution than DLIR-H across all conditions. As expected, TTF50% decreased with increasing phantom size, reflecting the impact of body habitus on image sharpness. ASIR-V also demonstrated slightly steeper slopes, particularly for the small phantom; however, the differences between ASIR-V and DLIR-H were less pronounced at low keV, in particular for small and large phantoms.

#### Noise

**Fig. 5:**
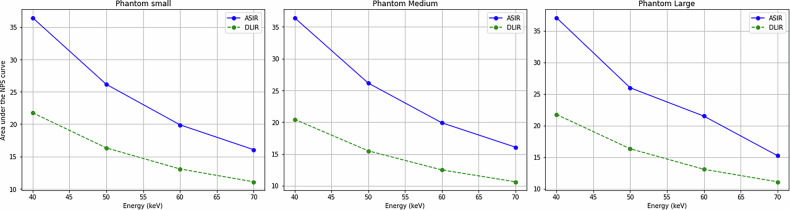
Comparison of noise amplitude between ASIR-V and DLIR across three phantom sizes (small, medium, large) at various energy levels (40–70 keV), expressed as the area under the noise power spectrum curve. ASIR, Adaptive statistical iterative reconstruction; DLIR, Deep learning image reconstruction

Noise amplitudes were evaluated by calculating the area under the noise power spectrum curve for each energy level, reconstruction algorithm, and phantom size (Fig. 5). As expected, image noise decreased with increasing VMI energy, with the highest noise observed at 40 keV, approximately twice that at 70 keV. DLIR-H significantly reduced noise compared to ASIR-V 50%, achieving a 44% reduction at 40 keV and a 33% reduction at 70 keV. Notably, the noise level at 40 keV with DLIR-H was comparable to that observed at 60 keV with ASIR-V 50% across all phantom sizes. Overall, noise levels remained consistent across the three phantom sizes.

#### Lesion detectability

**Fig. 6:**
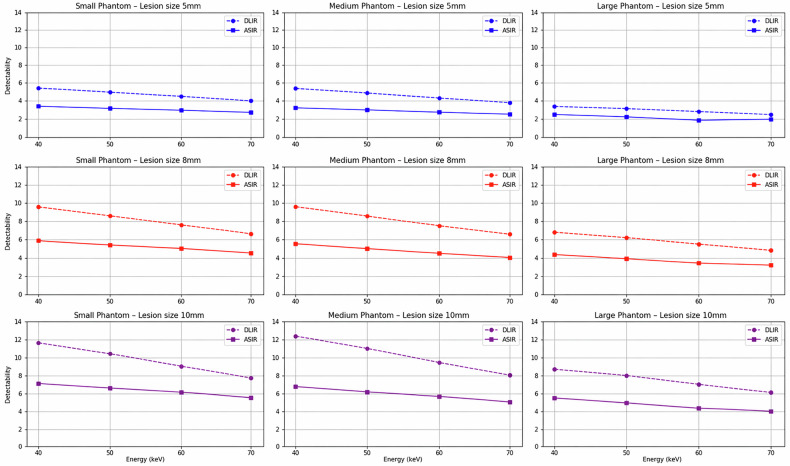
Detectability comparison between DLIR and ASIR-V across phantom sizes (small (S), medium (M), large (L)) and lesion sizes (5 mm, 8 mm, 10 mm) at varying monoenergetic levels (40–70 keV). ASIR, Adaptive statistical iterative reconstruction; DLIR, Deep learning image reconstruction

The detectability of simulated hypovascular lesions measuring 5, 8, and 10 mm in diameter is presented in Fig. 6 as a function of energy level, across different phantom sizes and reconstruction algorithms. Overall, detectability decreased with increasing VMI energy for all lesion sizes, phantom sizes, and reconstruction methods. As expected, larger lesion size was associated with higher detectability, while increased phantom size led to reduced detectability. All detectability values exceeded 1.89, indicating that the lesions were technically detectable across all conditions.

Maximum detectability was consistently observed at 40 keV. Across all lesion sizes, detectability declined as phantom size increased, reflecting the impact of simulated body habitus on lesion conspicuity. DLIR consistently outperformed ASIR-V for small- and medium-size phantoms, with markedly higher detectability scores across all lesion diameters. An improvement in lesion detectability was achieved when using DLIR compared with ASIR-V at 40 keV, with increases of 62% in the small-size phantom, 71% in the medium-size phantom, and 33% in the large-size phantom. Interestingly, detectability variation across energy levels diminished with decreasing lesion size and with increasing phantom size.

### Assessment of iodine load reduction potential

The potential for ICM reduction showed an inverse relationship with body habitus: lower BMI conditions were associated with greater opportunities for ICM reduction, whereas this potential decreased with increasing body size. For 5 mm lesions, the estimated theoretical potential of ICM reduction reached 41.9% in the medium phantom, 36.5% in the small-size phantom, and 31.3% in the large phantom. Across all tested conditions, including phantom size, lesion size, and energy level (keV), DLIR consistently enabled a greater theoretical potential for ICM reduction compared to ASIR-V. Moreover, larger lesion size was associated with increased ICM reduction potential, regardless of body size or reconstruction algorithm (Table 4).

**Table 4:** Lesion detectability according to lesion and phantom size across virtual monoenergetic reconstructions (40–70 keV), and detectability gain in percentage from 40 keV to 70 keV for deep learning image reconstruction

Phantom size	Lesion size (mm)	40 keV	50 keV	60 keV	70 keV	Detectability gain 40 *versus* 70 keV (%)
Small	5	5.47	5.01	4.53	4.01	36.5
	6	6.92	6.30	5.63	4.94	39.9
	8	9.51	8.60	7.57	6.55	45.2
	10	11.6	10.5	9.14	7.85	48.3
Medium	5	5.34	4.91	4.27	3.76	41.9
	6	6.99	6.38	5.52	4.81	45.1
	8	9.95	8.99	7.73	6.64	49.8
	10	12.4	11.1	9.55	8.14	52.7
Large	5	3.25	3.07	2.77	2.47	31.3
	6	4.45	4.17	3.72	3.30	35.0
	8	6.75	6.25	5.53	4.83	39.8
	10	8.75	8.07	7.11	6.14	42.4

## Discussion

This phantom-based study demonstrates that combining low-keV VMI with DLIR substantially enhances lesion detectability, supporting a theoretical potential for ICM reduction of up to 31.3% under phantom conditions.

In our study, VMI at 40 keV yielded a contrast approximately four times higher than that observed at 70 keV, regardless of phantom size, suggesting that this contrast gain is independent of body habitus. In line with findings by Cao et al and Pauthe et al [22, 23], no significant difference in contrast enhancement was observed between the iterative reconstruction algorithm and the DLIR. To better reflect clinical reality, the iodine concentrations used to simulate liver parenchyma and hypovascular metastases were derived directly from attenuation values measured in real patient CT examinations. While previous phantom studies typically used 2 mg I/mL for liver parenchyma [9, 24–26] and up to 1 mg I/mL for lesions [24], our data indicate that hypovascular metastases more realistically correspond to an iodine concentration of 0.45 mg I/mL. While ASIR-V mainly reduces noise magnitude and alters texture, DLIR more effectively lowers noise amplitude while preserving a texture closer to that of filtered back projection [7, 14]. This ability to balance image smoothness with diagnostic texture realism further supports the integration of DLIR into clinical low-keV protocols.

Contrast-dependent spatial resolution was found to be slightly higher with ASIR-V 50% compared to DLIR-H across all experimental conditions. However, the slight reduction in spatial resolution observed with DLIR-H did not compromise lesion detectability. This differs from the findings of Greffier et al [9], who reported marginally superior spatial resolution with DLIR-H using different specific experimental parameters such as lesion contrast, radiation dose, and phantom size. Moreover, the potential impact of the slightly reduced spatial resolution with DLIR-H on the detectability of very small lesions (< 5 mm) under *in vivo* conditions warrants further investigation, as these scenarios may be more sensitive to small differences in spatial resolution, particularly because human tissues exhibit greater heterogeneity than the controlled phantom conditions. Detectability was up to twice as high with DLIR compared to ASIR-V 50%, primarily due to the substantial noise reduction provided by DLIR. This advantage was particularly evident for 5 mm lesions in small- and medium-size phantoms, where detectability increased by approximately 40%. Our findings are in line with those of Zhong et al and Greffier et al [14, 26], who also reported superior detectability with the combination of 40 keV and DLIR-H. In large phantom, however, the benefit was attenuated, with an average improvement of around 26%, suggesting that the relative performance gain with DLIR may be limited by increased noise and photon starvation in larger body sizes. However, although the excellent detection performance across all conditions in our study, it is important to acknowledge the gap between these results and typical clinical practice. In clinical scenarios, the liver parenchyma and lesions exhibit more heterogeneity, with greater variability in both lesion characteristics and surrounding tissue. The sensitivity for detecting hypovascular liver lesions, particularly those smaller than 1 cm, is low with CT, especially for lesions around 5 mm. This inherent complexity can affect lesion conspicuity, making detection more challenging than what is observed in a phantom with controlled experimental conditions.

In this phantom study, we estimated a theoretical ICM reduction of 31.3% for the large phantom and 41.9% for the medium-sized phantom based on the improvement of detectability. Three studies employing circulation phantoms have demonstrated a theoretical potential to lower the required dose of ICM in CT angiography [19–21]. These investigations consistently observed a linear relationship between the contrast-to-noise ratio and different iodine concentrations for different energy levels or CT scanner types. Our study extends these findings by exploring the hypothetical potential of ICM reduction, comparing 70 keV reconstruction with 40 keV level. Unlike prior work, we employ the detectability index d′ instead of contrast-to-noise ratio, allowing us to account for both spatial resolution and object size, which provides a more realistic assessment of image quality. Furthermore, we evaluate the impact of variations in body habitus on the theoretical contrast reduction, an aspect not addressed in previous phantom or clinical studies, enhancing the clinical relevance of our findings.

It should be emphasized that these values represent estimates based on a detectability index obtained under controlled phantom conditions. Accordingly, they cannot yet be directly extrapolated to routine clinical practice. Rather, they should be considered as a rational, task-based reference framework to inform and guide the design of future prospective clinical validation studies.

Previous investigations, primarily conducted in vascular imaging protocols, have demonstrated that low VMI enables substantial reductions in ICM dose with contrast protocols selected empirically, up to 56% in coronary CT angiography [27–29], 65% in pulmonary angiography [30, 31], and 50% in lower-extremity angiography [32, 33], while preserving diagnostic image quality. In the context of oncologic imaging, however, the potential for ICM reduction is generally considered more limited, which may partly explain the relatively few studies specifically addressing this application. Nonetheless, contrast dose reduction remains clinically important in oncology, particularly given the repeated imaging required in surveillance and treatment monitoring. Encouraging results have already been reported in several studies directly comparing single-energy CT protocols with DECT acquisitions: Lee et al achieved a 30% ICM reduction [34–36]; Gulizia et al reported 44% [10]; and both Saleh et al and Sakabe et al described reductions of up to 50% [30, 31]. However, these studies often relied on arbitrarily selected contrast protocols and iodine delivery rates, without using a task-based or systematic optimization approach.

Nevertheless, these results underscore the importance of individualized imaging protocols in oncologic CT, particularly in the context of personalized medicine. In this regard, it is important to note that adipose tissue is metabolically less active and poorly perfused compared to solid organs and muscle, playing a limited role in the distribution and dilution of contrast media within the bloodstream [37]. As a result, dosing ICM solely based on total body weight may lead to an overestimation of the actual iodine requirement in overweight individuals. This notion is supported by a recent systematic review, which highlighted the clinical benefit of tailoring iodine dose based on lean body weight to minimize unnecessary ICM exposure, particularly in overweight and obese patients [38].

This study has several limitations. As a phantom-based study, our findings reflect a controlled, theoretical model of image acquisition and contrast dynamics. While this allows precise manipulation of variables such as lesion size and contrast concentration, it cannot fully replicate the biological complexity of human tissues. Thus, prospective *in vivo* studies are needed to confirm clinical applicability and safety, likely testing a more conservative reduction than suggested by the phantom study. Second, the relationship between body habitus and contrast enhancement was modeled as linear, which may oversimplify complex physiological variables such as hemodynamics and tissue perfusion. Furthermore, while the phantom setup allowed for excellent control over iodine concentration and imaging parameters, it does not fully replicate several factors encountered in clinical practice, such as complex hepatic vascular anatomy and variable lesion locations. In addition, the study did not differentiate between benign and malignant hypovascular lesions, focusing solely on detectability performance. These aspects should be considered when interpreting the translational relevance of our findings. Third, all experiments were performed on a single CT scanner from one manufacturer, potentially limiting the generalizability of the results. Validation across different scanner platforms and reconstruction algorithms is warranted. Lastly, only one radiation dose level was evaluated per BMI group; assessing the impact of dose modulation within each category could further inform strategies for optimizing both image quality and radiation exposure.

In conclusion, this phantom study demonstrates that low-keV VMI combined with DLIR-H significantly improves hypovascular lesion detectability across simulated body habitus. These detectability gains provide a quantitative basis for hypothesizing ICM dose reduction; however, such reduction was not directly tested and remains theoretical. Further studies incorporating variable iodine concentrations and clinical validation are required before contrast dose optimization can be implemented in practice.

## Supplementary information

**Additional File 1:** S1: Spatial resolution (TTF values at 50%) across VMI energy levels and reconstruction algorithms for different phantom sizes

## Data Availability

The datasets used and/or analyzed during the current study are available from the corresponding author on reasonable request.

## Abbreviations

ASIR-V: Adaptive statistical iterative reconstruction-V
BMI: Body mass index
CT: Computed tomography
DECT: Dual-energy CT
DLIR: Deep learning image reconstruction
DLIR-H: High-strength DLIR
ICM: Iodinated contrast media
TTF: Task-based transfer function
VMI: Virtual monoenergetic image
