# Supplementary material for: Optimization of hypovascular liver lesion detectability in dual-energy CT using deep learning image reconstruction: a phantom study for potential iodine dose reduction
Source: Eur Radiol Exp. 2026 Jul 1;10:104. doi: 10.1186/s41747-026-00759-2 (PMC13323749; doi:10.1186/s41747-026-00759-2)
Supplement: Supplementary file 1 — Additional File 1: S1: Spatial resolution (TTF values at 50%) across VMI energy levels and reconstruction algorithms for different phantom sizes [file 41747_2026_759_MOESM1_ESM.pdf]

Optimization of hypovascular liver lesion detectability in dual-energy CT using deep learning image reconstruction: a phantom study for potential iodine dose reduction

ELECTRONIC SUPPLEMENTARY MATERIAL

S1: Spatial resolution (TTF values at 50%) across VMI energy levels and reconstruction algorithms for different phantom sizes

	**Small phantom**		**Medium phantom**		**Large phantom**	
**VMI (keV)**	**ASIR-V**	**DLIR-H**	**ASIR-V**	**DLIR-H**	**ASIR-V**	**DLIR-H**
**40**	0.213	0.200	0.203	0.165	0.131	0.122
**50**	0.232	0.210	0.209	0.173	0.148	0.126
**60**	0.247	0.225	0.219	0.179	0.153	0.131
**70**	0.272	0.238	0.241	0.190	0.177	0.138

Note 1 Abbreviations: ASIR-V; adaptative statistical iterative reconstruction-V, DLIR-H; deep learning image reconstruction -High, VMI; virtual monochromatic image
